# In Vitro Combination Effect of Topical and Oral Anti-Onychomycosis Drugs on *Trichophyton rubrum* and *Trichophyton interdigitale*

**DOI:** 10.3390/jof7030208

**Published:** 2021-03-12

**Authors:** Keita Sugiura, Akane Masumoto, Haruki Tachibana, Yoshiyuki Tatsumi

**Affiliations:** Pharmacology Department, Drug Research Center, Kaken Pharmaceutical Co., Ltd., 14 Shinomiya, Minamigawara-cho, Yamashina-ku, Kyoto 607-8042, Japan; masumoto_akane@kaken.co.jp (A.M.); tachibana_haruki@kaken.co.jp (H.T.); tatsumi_yoshiyuki@kaken.co.jp (Y.T.)

**Keywords:** anti-onychomycosis drug, combination antifungal effect, FIC index

## Abstract

To evaluate the combination effects of anti-onychomycosis drugs, the minimum inhibitory concentrations of topical (efinaconazole, luliconazole, and tavaborole) and oral (itraconazole and terbinafine) drugs for *Trichophyton rubrum* and *Trichophyton interdigitale* (8 each, with a total of 16 strains) were determined using the microdilution checkerboard technique based on the Clinical and Laboratory Standard Institute guidelines. No antagonism was observed between the topical and oral drugs against all the tested strains. Efinaconazole with terbinafine exerted a synergistic effect on 43.8% of the strains tested (7/16 strains) and efinaconazole with itraconazole on 12.5% (2/16 strains). Conversely, luliconazole showed no synergistic effect with terbinafine but was synergistically effective with itraconazole against 31.3% of the strains (5/16 strains). Tavaborole showed no synergistic effect with terbinafine and was synergistically effective with itraconazole against 18.8% of the strains (3/16 strains). The results suggest that a combination of topical and oral drugs could be a potential clinical option for onychomycosis treatment, and overall, the efinaconazole and oral drug combination would be the most advantageous among the tested combinations.

## 1. Introduction

Onychomycosis, a common fungal nail infection, is mainly caused by *Trichophyton rubrum* (*T. rubrum*) and *Trichophyton interdigitale* (*T. interdigitale*) in the nail plate and bed, with the prevalence estimated to be between 10% (Japan) and 13.8% (United States of America) [1,2]. Onychomycosis is difficult to cure, impacting the patient’s quality of life by resulting in walking difficulties and poor nail appearance, and can be a source of secondary infection or spread to other family members [3].

Oral terbinafine and itraconazole are the preferred treatment options for onychomycosis [4,5]. However, their use is limited by hepatotoxicity and drug–drug interactions (especially with itraconazole), representing a safety concern particularly in older persons, in whom an underlying disease and polypharmacy are common [6]. In recent years, three new topical antifungals (efinaconazole, luliconazole, and tavaborole) have been launched in North America and/or Japan, which have a low risk of inducing hepatotoxic side effects and drug–drug interactions; however, their complete cure rates are generally lower than those of oral drugs [7].

The failure rate of oral antifungal therapy for onychomycosis is 20%, with a high recurrence rate of 10–53% [8,9,10,11,12]. Some factors also contributing to unsuccessful therapy are the patient’s susceptibility, pattern of resistant fungal growth, presence of fungal dormant spores in the nail, low bioavailability of the drug, and lack of drug penetration into the nail [8,9,13]. Therefore, many treatment methods have been introduced to overcome the limitations of onychomycosis therapy, including a combination of oral and topical antifungals or a combination of two oral antifungals [8,14]. In addition, because the elimination half-life of orally administered terbinafine and itraconazole from nails is long, ranging from 24 to 156 day and 32 to 147 days, respectively [15], switch therapy involving switching from oral to topical treatment may also be worth considering.

Dermatophytes reside in the nail plate and bed. Therefore, a drug’s entry route into infected nail sites plays a vital role in determining its efficacy. Oral drugs reach the nail bed by increasing antifungal levels in the bloodstream to levels that are in excess of the minimum inhibitory concentration (MIC). The primary route of drug delivery for topical drugs is transungual, where the drug is applied to the dorsal aspect of the nail plate, and it then penetrates the underlying nail bed [16]. Therefore, combination therapy involves two-way penetration of the nail bed by oral drugs and of the nail plate by topical drugs, providing a higher cure rate compared to that provided by each monotherapy, presumably because of a synergistic antifungal effect and diffusion of the two drugs at an effective concentration in the nail. Moreover, combination therapy with oral (terbinafine and itraconazole) and topical (amorolfine and ciclopirox) antifungals has been shown to lead to improvement in mycological and clinical outcomes, reduced therapy duration, and minimized risk of side effects due to systemic treatment [8,9,14,17].

Compared to amorolfine and ciclopirox, the three new topical drugs (efinaconazole, tavaborole, and luliconazole) have been reported to possess higher nail permeability or efficacy coefficients, which are calculated using nail permeability and antifungal activity in the presence of keratin [18,19]. To improve the existing onychomycosis treatment method in terms of therapeutic effect and recurrence reduction, the new topical antifungals would be worth investigating for their use with oral antifungals in combination therapy. However, there are no reports on combination therapy with these new topical and oral antifungals for onychomycosis treatment. Therefore, in this study, to obtain information about future combination therapy, these topical drugs were investigated for their in vitro combination effects with oral drugs against *T. rubrum* and *T. interdigitale*.

## 2. Materials and Methods

### 2.1. Strains

This study included eight strains of *T. rubrum* (NBRC 5807, NBRC 6203, NBRC 9185, IFM 46636, IFM 47615, IFM 47624, IFM 47625, and IFM 46157) and eight strains of *T. interdigitale* (IFM 62762, IFM 62959, IFM 63291, IFM 63319, IFM 63830, IFM 64133, IFM 64134, and IFM 64902). The NBRC and IFM strains were provided by the National Institute of Technology and Evaluation and Medical Mycology Research Center, Chiba University, respectively. All the strains were clinical isolates.

### 2.2. Antifungal Drugs

Efinaconazole and itraconazole were purchased from Sigma-Aldrich Co., LLC. (St. Louis, MO, USA); terbinafine hydrochloride from Tokyo Chemical Industry Co., Ltd. (Tokyo, Japan); and luliconazole and tavaborole from Toronto Research Chemicals Inc. (Toronto, ON, Canada).

### 2.3. Media

Potato dextrose agar was purchased from Nissui Pharmaceutical Co., Ltd. (Tokyo, Japan). 3-(N-morpholino) propanesulfonic acid (Nacalai Tesque, Inc., Tokyo, Japan)-buffered Roswell Park Memorial Institute 1640 medium (Nissui Pharmaceutical Co., Ltd.), pH 7.0, was used in this study.

### 2.4. Minimum Inhibitory Concentration (MIC) Test

The 16 test strains were subcultured on potato dextrose agar plates and grown at 30 °C for 7 days. From the subcultures, fungal conidia were collected in 0.85% saline. The conidial suspensions were filtered using a cell strainer (mesh size: 40 µm). The fungal concentration was adjusted to 4 × 10^3^ cells/mL (twice the final fungal concentration). The MIC test was performed using the broth microdilution method referred to in the Clinical and Laboratory Standard Institute protocol M38 [20]. Serial two-fold dilutions of the antifungal drugs were prepared in the 3-(N-morpholino) propanesulfonic acid-buffered Roswell Park Memorial Institute 1640 medium (pH 7.0). To calculate the fractional inhibitory concentration (FIC) index, checkerboards were designed with efinaconazole (0.000061–0.063 µg/mL) and terbinafine (0.00024–0.25 µg/mL), luliconazole (0.000015–0.016 µg/mL) and terbinafine (0.00024–0.25 µg/mL), tavaborole (0.016–16 µg/mL) and terbinafine (0.00024–0.25 µg/mL), efinaconazole (0.000061–0.063 µg/mL) and itraconazole (0.00098–1.0 µg/mL), luliconazole (0.000015–0.016 µg/mL) and itraconazole (0.00098–1.0 µg/mL), and tavaborole (0.016–16 µg/mL) and itraconazole (0.00098–1.0 µg/mL). Fifty µL dilutions of each test drug were added to round-bottom 96-well microplates. Then, 100 µL of an inoculum suspension was added. The microplates were incubated at 35 °C for 4 days. After the incubation, fungal growth was observed. The degree of fungal growth was assessed visually and graded: score 0, optically clear or absence of growth; score 1, slight growth or ~20% of the growth control; score 2, prominent reduction in growth or ~50% of the growth control; score 3, slight reduction in growth; score 4, no reduction in growth. MICs (for individual drugs and drugs in combination) were determined as the minimum concentrations required to inhibit 80% or more growth (scores 0 and 1) in a drug-free control well.

### 2.5. Calculation of the Fractional Inhibitory Concentration (FIC) Indexes and Evaluation of Drug Interactions

To evaluate drug interactions, the FIC indexes were calculated. The FIC index of the antifungal drugs (drugs A and B) was calculated using the following formula: (MIC of A in combination with B/MIC of A alone + MIC of B in combination with A/MIC of B alone) [21,22]. The interaction was considered synergistic if the FIC index was ≤0.50, additive if >0.50 but <1.0, indifferent if ≥1.0 but ≤2.0, and antagonistic if >2.0 [22].

## 3. Results

**Figure 1:**
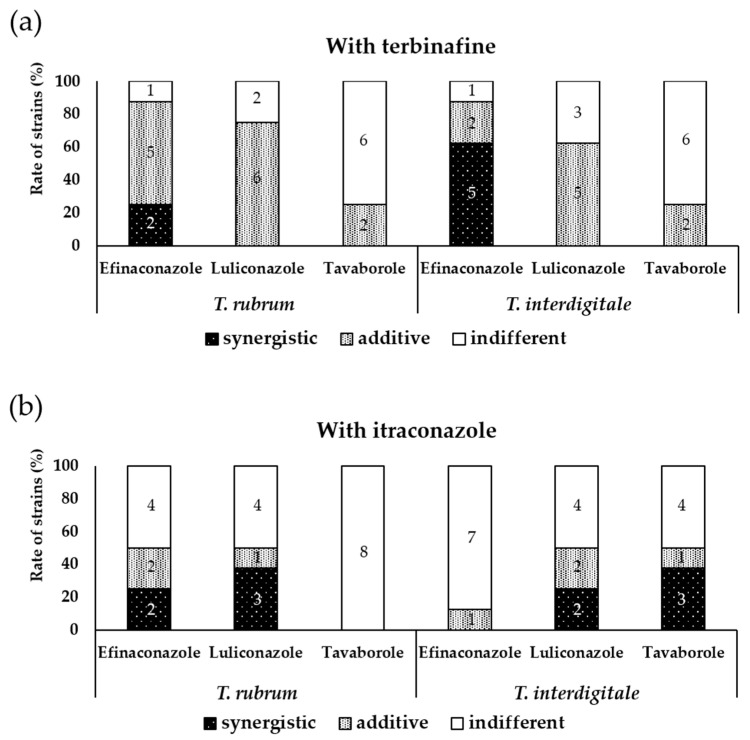
Combination antifungal effect of topical drugs and oral drugs against *T. rubrum* and *T. interdigitale* (each 8 strains) (**a**) with terbinafine, (**b**) with itraconazole. Figures in the bar express number of strains observed in each combination effect.

The efinaconazole and terbinafine combination exerted a synergistic effect on 43.8% (7/16) of *T. rubrum* and *T. interdigitale* strains, with an FIC index range of 0.19–0.50 (Table 1 and Table 2 and Figure 1). The efinaconazole and itraconazole combination showed no synergistic effect on all *T. interdigitale* strains, but exhibited a synergistic effect on 25.0% (2/8) of *T. rubrum* strains, with an FIC index range of 0.31–0.38 (Table 3 and Table 4 and Figure 1). On the other hand, the luliconazole and terbinafine combination showed no synergistic effect on all 16 strains (Table 1 and Table 2 and Figure 1), whereas the luliconazole and itraconazole combination had a synergistic effect on 31.3% (5/16) of the strains, with an FIC index range of 0.31–0.38 (Table 3 and Table 4 and Figure 1). The tavaborole and terbinafine combination showed no synergistic effect on all 16 strains (Table 1 and Table 2 and Figure 1). The tavaborole and itraconazole combination showed no synergistic effect on all *T. rubrum* strains but showed a synergistic effect on 37.5% (3/8) of *T. interdigitale* strains, with an FIC index range of 0.09–0.38. Moreover, the combination of luliconazole or tavaborole with oral antifungals showed an indifferent effect on some strains, with a relatively high FIC index of 2.00 (Table 3 and Table 4 and Figure 1).

## 4. Discussion

Topical (amorolfine, ciclopirox, efinaconazole, tavaborole, and luliconazole) and oral antifungals (terbinafine and itraconazole) are currently used for onychomycosis treatment. Azole antifungals (triazole class: efinaconazole and itraconazole; imidazole class: luliconazole), terbinafine, and amorolfine inhibit lanosterol 14α-demethylase, squalene epoxidase, and Δ14 reductase/Δ7-8 isomerase, respectively. These antifungals consequentially block ergosterol biosynthesis in fungal cells [23,24]. Ciclopirox chelates polyvalent cations, such as Fe^3+^ and Al^3+^, resulting in the inhibition of metal-dependent enzymes responsible for degrading peroxides inside fungal cells [23]. Tavaborole inhibits leucyl-tRNA synthetase and consequentially blocks protein synthesis in fungal cells [23].

The in vitro combined effect of topical (amorolfine and ciclopirox) and oral drugs on dermatophytes has been reported [22,25,26]. Using the checkerboard method, Harman et al. reported synergistic effects (FIC index ≤ 1) of amorolfine and terbinafine as well as amorolfine and itraconazole combinations in 29% and 50% of 10 dermatophytes strains, respectively [25]. Tamura et al. also reported that the amorolfine and itraconazole combination had a synergistic effect (FIC index ≤ 0.5) on 32% (6/19) of *T. rubrum* and *T. interdigitale* strains, with an FIC index range of 0.24–0.49 [26]. Santos et al. reported a synergistic effect of ciclopirox and itraconazole on two strains of *T. rubrum* and *T. interdigitale* [22]. In clinical research, combination therapies with topical (amorolfine or ciclopirox) and oral drugs have been reported to have a greater effect than each onychomycosis monotherapy [14,27,28]. The enhanced therapeutic effects are partially attributed to the combined antifungal effects in addition to the merit of the two-way penetration of the nail bed and plate.

Because there are no reports on the combination of the recently developed topical drugs and existing oral drugs, the in vitro antifungal activity of the topical drugs (efinaconazole, tavaborole, and luliconazole) in combination with oral drugs (terbinafine and itraconazole) against dermatophytes was evaluated in the present study. The topical and oral antifungal combination did not show an antagonistic effect on the tested strains, suggesting that these combinations and switch therapies could be potential clinical options for onychomycosis treatment. Furthermore, since two-way nail penetration is generally effective for onychomycosis treatment, the in vitro indifferent effects in a few cases might not have a bad influence on the efficacy of these combinations. In addition, in combination with terbinafine, only efinaconazole exerted synergistic effects against both dermatophytes species investigated in this study among the topical drugs. In combination with itraconazole, luliconazole and efinaconazole were synergistically effective against *T. rubrum* to the same extent. On the other hand, against *T. interdigitale*, luliconazole and tavaborole were synergistically effective with itraconazole. Considering *T. rubrum* as the main causative fungi of onychomycosis [29], these results comprehensively suggest that efinaconazole is the most advantageous drug among the tested topical antifungals for combination therapy with oral antifungals for onychomycosis treatment.

It is worth noting that efinaconazole in combination with terbinafine showed synergistic effects on the dermatophyte strains, which is unlikely with tavaborole and luliconazole. There are no reports on the in vitro combination effects of other triazoles (e.g., itraconazole and voriconazole) and terbinafine on dermatophytes. Conversely, combinations of triazoles (itraconazole or voriconazole) and terbinafine have been reported to show synergistic effects on *Aspergillus*, *Fusarium*, and *Candida* species [30,31,32,33,34]. Although the oral triazoles in combination with terbinafine may also show a synergistic effect on dermatophytes, they cannot exert an in vivo synergistic effect with oral terbinafine via two-way nail penetration, because they are not topically administered for onychomycosis treatment.

Although several studies have examined the synergistic effects of antifungals [35,36], few have provided explanations for the mechanisms of drug synergy [26,37]. Although we do not have a clear explanation for the mechanism of the combination effect of efinaconazole and terbinafine, the mechanism of synergy may be attributed to the blockage of ergosterol biosynthesis at different levels, as suggested in the mechanism of the synergistic effect of voriconazole and terbinafine on *Candida albicans* [34]. Moreover, although luliconazole, efinaconazole, and itraconazole inhibit lanosterol 14α-demethylase, luliconazole did not show a synergistic effect with terbinafine, which is unlikely with efinaconazole, and it also showed a synergistic effect with itraconazole on more strains than efinaconazole. This suggests that luliconazole (imidazole class) may have a mechanism involving a combination action, different from the triazole class, such as efinaconazole and itraconazole. To elucidate the mechanism of the synergistic effect of the efinaconazole and terbinafine combination on dermatophytes, including the difference between triazoles and luliconazole, we need to assess cellular and molecular changes after exposing fungi to the drug alone or in combination with terbinafine.

The present study suggested that no antagonistic effects were observed in all the tested strains when a combination of the topical and oral drugs was used, implying their possible use in combination and switch therapy. Overall, among the three topical drugs, the combination effects of efinaconazole and oral drugs on dermatophytes was the most advantageous for such therapeutic strategies. Clinical studies are warranted to elucidate the potential utility of combination therapy with the topical and oral antifungals for onychomycosis treatment.

## Figures and Tables

**Table 1:** Antifungal effect of the combination of topical drugs and terbinafine on *T. rubrum.*

Topical Drugs	Strain No.	MIC of Topical Drug (µg/mL)				MIC of Terbinafine (µg/mL)				FIC Index	Drug Interaction
		Alone	With Terbinafine			Alone	With Topical Drug				
Efinaconazole	NBRC 5807	0.0020	0.00012	−	0.0020	0.031	0.0039	−	0.016	0.19	Synergistic
	NBRC 6203	0.0020	0.00012	−	0.0020	0.0078	0.0020	−	0.0078	0.31	Synergistic
	NBRC 9185	0.0039	0.00098	−	0.0039	0.0078	0.0078			1.25	Indifferent
	IFM 46636	0.0039	0.00012	−	0.0039	0.031	0.016	−	0.031	0.53	Additive
	IFM 47615	0.0078	0.00049	−	0.0078	0.016	0.0078	−	0.016	0.56	Additive
	IFM 47624	0.0039	0.00098	−	0.0039	0.0078	0.0039	−	0.0078	0.75	Additive
	IFM 47625	0.0039	0.00024	−	0.0039	0.016	0.0078	−	0.016	0.56	Additive
	IFM 46157	0.0039	0.0020	−	0.0039	0.0078	0.0020	−	0.0078	0.75	Additive
							FIC index range			0.19–1.25	
						Geometric mean FIC index				0.54	
Luliconazole	NBRC 5807	0.00024	0.000031	−	0.00024	0.016	0.0078	−	0.016	0.63	Additive
	NBRC 6203	0.00024	0.00012	−	0.00024	0.0078	0.0020	−	0.0078	0.75	Additive
	NBRC 9185	0.00024	0.000061	−	0.00024	0.0078	0.0039	−	0.0078	0.75	Additive
	IFM 46636	0.00024	0.000061	−	0.00024	0.016	0.016			1.25	Indifferent
	IFM 47615	0.00049	0.00024	−	0.00049	0.016	0.0039	−	0.016	0.75	Additive
	IFM 47624	0.00024	0.00012	−	0.00024	0.0078	0.0039	−	0.0078	1.00	Indifferent
	IFM 47625	0.00024	0.000061	−	0.00024	0.016	0.0078	−	0.016	0.75	Additive
	IFM 46157	0.00049	0.00012	−	0.00024	0.0078	0.0039	−	0.0078	0.75	Additive
							FIC index range			0.63–1.25	
						Geometric mean FIC index				0.81	
Tavaborole	NBRC 5807	4.0	0.50	−	4.0	0.031	0.016			0.63	Additive
	NBRC 6203	4.0	0.063	−	4.0	0.0078	0.0078			1.02	Indifferent
	NBRC 9185	4.0	0.50	−	4.0	0.0078	0.0078			1.13	Indifferent
	IFM 46636	4.0	2.0	−	4.0	0.031	0.016	−	0.031	1.00	Indifferent
	IFM 47615	4.0	4.0			0.016	0.016			2.00	Indifferent
	IFM 47624	4.0	4.0			0.0078	0.0078			2.00	Indifferent
	IFM 47625	4.0	1.0	−	4.0	0.031	0.016	−	0.031	0.75	Additive
	IFM 46157	4.0	2.0	−	4.0	0.0078	0.0078			1.50	Indifferent
							FIC index range			0.63–2.00	
						Geometric mean FIC index				1.16	

MIC: mic minimum inhibitory concentration, FIC: Fractional Inhibitory Concentration. NBRC: National Institute of Technology and Evaluation Biological Resource Center, IFM: Institute of Food-Microbiology Chiba Medical College.

**Table 2:** Antifungal effect of the combination of topical drugs and terbinafine on *T. interdigitale.*

Topical Drugs	Strain No.	MIC of Topical Drug (µg/mL)				MIC of Terbinafine (µg/mL)				FIC Index	Drug Interaction
		Alone	With Terbinafine			Alone	With Topical Drug				
Efinaconazole	IFM 62762	0.0078	0.0020	−	0.0078	0.0078	0.0039	−	0.0078	0.75	Additive
	IFM 62959	0.0078	0.00098	−	0.0039	0.0078	0.0020	−	0.0078	0.38	Synergistic
	IFM 63291	0.0078	0.0020	−	0.0039	0.0078	0.0020	−	0.0078	0.50	Synergistic
	IFM 63319	0.0078	0.00049	−	0.0078	0.016	0.0078	−	0.016	0.56	Additive
	IFM 63830	0.00098	0.00012	−	0.00098	0.016	0.0039	−	0.016	0.38	Synergistic
	IFM 64133	0.0078	0.00049	−	0.0078	0.031	0.0078	−	0.031	0.31	Synergistic
	IFM 64134	0.0078	0.00098	−	0.0078	0.031	0.0078	−	0.031	0.38	Synergistic
	IFM 64902	0.0020	0.00098	−	0.0020	0.016	0.016			1.50	Indifferent
							FIC index range			0.31–1.50	
						Geometric mean FIC index				0.52	
Luliconazole	IFM 62762	0.00024	0.000061	−	0.00024	0.0078	0.0039	−	0.0078	0.75	Additive
	IFM 62959	0.00024	0.00012	−	0.00024	0.0078	0.0020	−	0.0078	0.75	Additive
	IFM 63291	0.00024	0.00012	−	0.00024	0.0078	0.0039	−	0.0078	1.00	Indifferent
	IFM 63319	0.00049	0.000061	−	0.00049	0.016	0.0078	−	0.016	0.63	Additive
	IFM 63830	0.00012	0.000061	−	0.00012	0.016	0.0039	−	0.016	0.75	Additive
	IFM 64133	0.00024	0.000061	−	0.00024	0.031	0.016	−	0.031	0.75	Additive
	IFM 64134	0.00024	0.00012	−	0.00024	0.031	0.016	−	0.031	1.00	Indifferent
	IFM 64902	0.00012	0.00012			0.0078	0.0078			2.00	Indifferent
							FIC index range			0.63–2.00	
						Geometric mean FIC index				0.89	
Tavaborole	IFM 62762	4.0	0.50	−	4.0	0.0078	0.0078			1.13	Indifferent
	IFM 62959	4.0	2.0			0.0078	0.0078			1.50	Indifferent
	IFM 63291	4.0	2.0	−	4.0	0.0078	0.0078			1.50	Indifferent
	IFM 63319	8.0	4.0			0.016	0.00098	−	0.016	0.56	Additive
	IFM 63830	4.0	2.0	−	4.0	0.016	0.016			1.50	Indifferent
	IFM 64133	4.0	1.0	−	4.0	0.031	0.016	−	0.031	0.75	Additive
	IFM 64134	4.0	4.0			0.031	0.016	−	0.031	1.50	Indifferent
	IFM 64902	8.0	1.0	−	8.0	0.0078	0.0078			1.13	Indifferent
							FIC index range			0.56–1.50	
						Geometric mean FIC index				1.13	

MIC: mic minimum inhibitory concentration, FIC: Fractional Inhibitory Concentration. IFM: Institute of Food-Microbiology Chiba Medical College.

**Table 3:** Antifungal effect of the combination of topical drugs and itraconazole on *T. rubrum.*

Topical Drugs	Strain No.	MIC of Topical Drug (µg/mL)				MIC of Itraconazole (µg/mL)				FIC Index	Drug Interaction
		Alone	With Itraconazole			Alone	With Topical Drug				
Efinaconazole	NBRC 5807	0.0020	0.00049	−	0.0020	0.016	0.016			1.25	Indifferent
	NBRC 6203	0.0020	0.00012	−	0.00098	0.016	0.0039	−	0.016	0.31	Synergistic
	NBRC 9185	0.0039	0.0020	−	0.0039	0.016	0.016			1.50	Indifferent
	IFM 46636	0.0039	0.00049	−	0.0039	0.016	0.0039	−	0.016	0.38	Synergistic
	IFM 47615	0.0078	0.0039	−	0.0078	0.031	0.016	−	0.031	1.00	Indifferent
	IFM 47624	0.0039	0.00098	−	0.0039	0.016	0.0078	−	0.016	0.75	Additive
	IFM 47625	0.0039	0.00049	−	0.0039	0.063	0.031	−	0.063	0.63	Additive
	IFM 46157	0.0039	0.00024	−	0.0039	0.031	0.031			1.06	Indifferent
							FIC index range			0.31–1.50	
						Geometric mean FIC index				0.76	
Luliconazole	NBRC 5807	0.00024	0.000031	−	0.00012	0.016	0.0039	−	0.016	0.38	Synergistic
	NBRC 6203	0.00024	0.000031	−	0.00024	0.016	0.0039	−	0.0078	0.38	Synergistic
	NBRC 9185	0.00024	0.00024			0.016	0.016			2.00	Indifferent
	IFM 46636	0.00024	0.00012	−	0.00024	0.016	0.0039	−	0.016	0.75	Additive
	IFM 47615	0.00049	0.00024	−	0.00049	0.031	0.016	−	0.031	1.00	Indifferent
	IFM 47624	0.00024	0.000061	−	0.00024	0.016	0.016			1.25	Indifferent
	IFM 47625	0.00024	0.00024			0.016	0.016			2.00	Indifferent
	IFM 46157	0.00024	0.000031	−	0.00024	0.031	0.0078	−	0.016	0.38	Synergistic
							FIC index range			0.38–2.00	
						Geometric mean FIC index				0.82	
Tavaborole	NBRC 5807	4.0	4.0			0.016	0.0078	−	0.016	1.50	Indifferent
	NBRC 6203	4.0	2.0	−	4.0	0.0078	0.0078			1.50	Indifferent
	NBRC 9185	4.0	0.13	−	4.0	0.016	0.016			1.03	Indifferent
	IFM 46636	4.0	0.13	−	4.0	0.016	0.016			1.03	Indifferent
	IFM 47615	4.0	4.0			0.031	0.031			2.00	Indifferent
	IFM 47624	4.0	1.0	−	4.0	0.016	0.016			1.25	Indifferent
	IFM 47625	4.0	2.0	−	4.0	0.016	0.016			1.50	Indifferent
	IFM 46157	4.0	2.0	−	4.0	0.016	0.0078	−	0.016	1.00	Indifferent
							FIC index range			1.00–2.00	
						Geometric mean FIC index				1.32	

MIC: mic minimum inhibitory concentration, FIC: Fractional Inhibitory Concentration. NBRC: National Institute of Technology and Evaluation Biological Resource Center, IFM: Institute of Food-Microbiology Chiba Medical College.

**Table 4:** Antifungal effect of the combination of topical drugs and itraconazole on *T. interdigitale.*

Topical Drugs	Strain No.	MIC of Topical Drug (µg/mL)				MIC of Itraconazole (µg/mL)				FIC Index	Drug Interaction
		Alone	With Itraconazole			Alone	With Topical Drug				
Efinaconazole	IFM 62762	0.0020	0.00098	−	0.0020	0.031	0.031			1.50	Indifferent
	IFM 62959	0.0039	0.00024	−	0.0039	0.031	0.031			1.06	Indifferent
	IFM 63291	0.0039	0.0020	−	0.0039	0.031	0.031			1.50	Indifferent
	IFM 63319	0.0078	0.0039	−	0.0078	0.031	0.0039	−	0.031	0.63	Additive
	IFM 63830	0.00098	0.00049	−	0.00098	0.0039	0.0039			1.50	Indifferent
	IFM 64133	0.016	0.0078	−	0.016	0.031	0.016	−	0.031	1.00	Indifferent
	IFM 64134	0.0078	0.0078			0.016	0.0078	−	0.016	1.50	Indifferent
	IFM 64902	0.0078	0.0078			0.031	0.016	−	0.031	1.50	Indifferent
							FIC index range			0.63–1.50	
						Geometric mean FIC index				1.22	
Luliconazole	IFM 62762	0.00024	0.000031	−	0.00024	0.031	0.0078	−	0.016	0.38	Synergistic
	IFM 62959	0.00049	0.000031	−	0.00049	0.031	0.0078	−	0.031	0.31	Synergistic
	IFM 63291	0.00024	0.00012	−	0.00024	0.031	0.031			1.50	Indifferent
	IFM 63319	0.00049	0.00024	−	0.00049	0.031	0.0078	−	0.031	0.75	Additive
	IFM 63830	0.00012	0.000061			0.0039	0.0020	−	0.0039	1.00	Indifferent
	IFM 64133	0.00024	0.00012	−	0.00024	0.031	0.0078	−	0.031	0.75	Additive
	IFM 64134	0.00024	0.00012	−	0.00024	0.0078	0.0078			1.50	Indifferent
	IFM 64902	0.00024	0.00024			0.031	0.016	−	0.031	1.50	Indifferent
							FIC index range			0.31–1.50	
						Geometric mean FIC index				0.83	
Tavaborole	IFM 62762	4.0	0.50	−	2.0	0.031	0.0078	−	0.016	0.38	Synergistic
	IFM 62959	4.0	0.13	−	2.0	0.031	0.0020	−	0.016	0.09	Synergistic
	IFM 63291	4.0	2.0	−	4.0	0.031	0.0078	−	0.016	0.75	Additive
	IFM 63319	4.0	0.063	−	4.0	0.031	0.0078	−	0.031	0.27	Synergistic
	IFM 63830	4.0	2.0	−	4.0	0.0039	0.0039			1.50	Indifferent
	IFM 64133	4.0	2.0	−	4.0	0.031	0.016	−	0.031	1.00	Indifferent
	IFM 64134	4.0	2.0	−	4.0	0.031	0.031			1.50	Indifferent
	IFM 64902	2.0	1.0	−	2.0	0.031	0.016	−	0.031	1.00	Indifferent
							FIC index range			0.09–1.50	
						Geometric mean FIC index				0.60	

MIC: mic minimum inhibitory concentration, FIC: Fractional Inhibitory Concentration. IFM: Institute of Food-Microbiology Chiba Medical College.
